# 6,6′-Dihydr­oxy-2,2′-[(pentane-1,5-diyl­dioxy)bis­(nitrilo­methyl­idyne)]diphenol

**DOI:** 10.1107/S1600536808023064

**Published:** 2008-07-26

**Authors:** Wen-Kui Dong, Xue-Ni He, Yong-Hong Guan, Li Xu, Zong-Li Ren

**Affiliations:** a School of Chemical and Biological Engineering, Lanzhou Jiaotong University, Lanzhou 730070, People’s Republic of China

## Abstract

The mol­ecule of the title compound, C_19_H_22_N_2_O_6_, assumes a W-shaped configuration with the dihedral angle between the two halves of the mol­ecule being 82.48 (5)°. There is one half-mol­ecule in the asymmetric unit with a crystallographic twofold rotation axis passing through the central C atom of the five methylene groups in the [—CH=N—O—(CH_2_)_5_—O—N=CH—] bridge. The dihedral angle formed by the two benzene rings in each mol­ecule of the title compound is 84.18 (4)°. There are strong intra­molecular O—H⋯N and O—H⋯O hydrogen bonds and weak inter­molecular π–π stacking inter­actions between neighbouring benzene rings, and the inter­molecular plane-to-plane distances are 3.488 (2) and 3.841 (3) Å along the *b* and *c* axes, respectively. In the crystal structure, inter­molecular O—H⋯O hydrogen bonds link each mol­ecule to two others, forming an infinite three-dimensional supra­molecular structure.

## Related literature

For related literature, see: Akine *et al.* (2001 ▶, 2005 ▶, 2006 ▶); Atwood (1997 ▶); Dong & Feng (2006 ▶); Dong, Zhao *et al.* (2008 ▶); Dong, He *et al.* (2008 ▶); Duan *et al.* (2007 ▶); Venkataramanan *et al.* (2005 ▶); Yu *et al.* (2008 ▶).
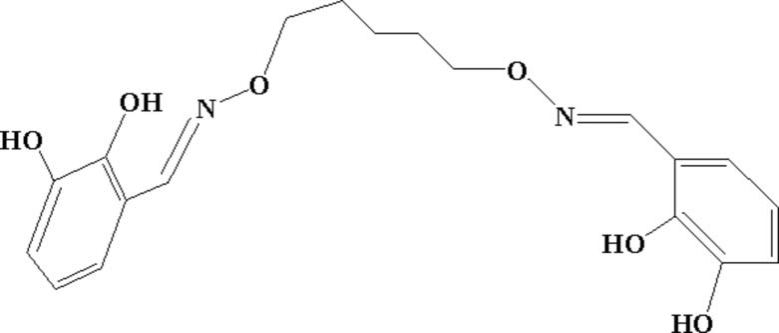


## Experimental

### 

#### Crystal data

C_19_H_22_N_2_O_6_
*M* *_r_* = 374.39
Monoclinic, 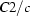

*a* = 28.439 (3) Å
*b* = 4.6997 (6) Å
*c* = 14.0843 (17) Å
β = 100.354 (2)°
*V* = 1851.8 (4) Å^3^
*Z* = 4
Mo *K*α radiation
μ = 0.10 mm^−1^
*T* = 298 (2) K
0.46 × 0.27 × 0.25 mm

#### Data collection

Siemens SMART 1000 CCD area-detector diffractometer
Absorption correction: multi-scan (*SADABS*; Sheldrick, 1996 ▶) *T*_min_ = 0.955, *T*_max_ = 0.975
4246 measured reflections
1621 independent reflections
837 reflections with *I* > 2σ(*I*)
*R*_int_ = 0.062

#### Refinement

*R*[*F* ^2^ > 2σ(*F* ^2^)] = 0.049
*wR*(*F* ^2^) = 0.135
*S* = 1.00
1621 reflections
123 parameters
H-atom parameters constrained
Δρ_max_ = 0.24 e Å^−3^
Δρ_min_ = −0.24 e Å^−3^

### 

Data collection: *SMART* (Siemens, 1996 ▶); cell refinement: *SMART*; data reduction: *SAINT* (Siemens, 1996 ▶); program(s) used to solve structure: *SHELXS97* (Sheldrick, 2008 ▶); program(s) used to refine structure: *SHELXL97* (Sheldrick, 2008 ▶); molecular graphics: *SHELXTL* (Sheldrick, 2008 ▶); software used to prepare material for publication: *SHELXTL*.

## Supplementary Material

Crystal structure: contains datablocks global, I. DOI: 10.1107/S1600536808023064/hg2427sup1.cif

Structure factors: contains datablocks I. DOI: 10.1107/S1600536808023064/hg2427Isup2.hkl

Additional supplementary materials:  crystallographic information; 3D view; checkCIF report

## Figures and Tables

**Table 1:** Hydrogen-bond geometry (Å, °)

*D*—H⋯*A*	*D*—H	H⋯*A*	*D*⋯*A*	*D*—H⋯*A*
O2—H2⋯N1	0.82	1.92	2.630 (3)	144
O3—H3⋯O2	0.82	2.24	2.689 (3)	115
O3—H3⋯O1^i^	0.82	2.29	2.958 (3)	139

Symmetry code: (i) 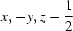.

**Fig. 1.**
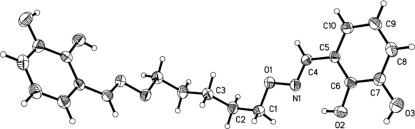
The molecular structure of the title compound with atom numbering scheme [Symmetry codes: -x + 1, y, -z + 3/2]. Displacement ellipsoids for non-hydrogen atoms are drawn at the 30% probability level.

**Fig. 2.**
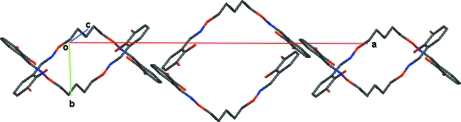
The W shape configuration of the title compound.

**Fig. 3.**
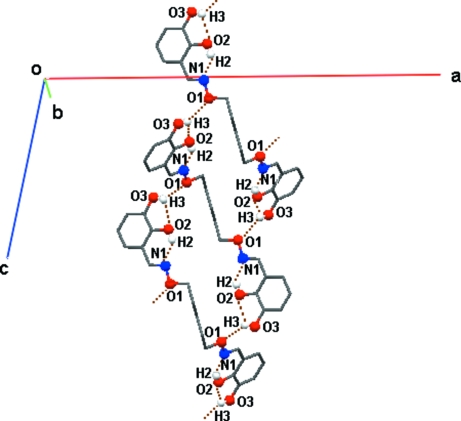
Part of the supramolecular structure of the title compound along b axis. Intra- and intermolecular hydrogen bonds are shown as dashed lines.

## supplementary crystallographic information

### Comment

Salen-type compounds are one of most versatile mixed-donor ligands in the field of coordination chemistry. There has been growing interest in salen-type ligands, mainly because of their wide application in the field of synthesis, biochemistry, photochemistry and catalysis (Akine *et al.*, 2006; Atwood, 1997; Yu *et al.*, 2008; Venkataramanan *et al.*, 2005). Many salen-type complexes have been structurally characterized (Akine *et al.*, 2006; Yu *et al.*, 2008), but only a relatively small number of free salen-type compounds have been characterized (Akine *et al.*, 2001). As an extension of our work (Dong & Feng, 2006; Duan *et al.*, 2007; Dong, Zhao *et al.*, 2008; Akine *et al.*, 2005) on the structural characterization of salen-type bisoxime compounds, the title compound, (Fig. 1), is reported here.

The molecule assumes a W shape with the dihedral angle between the two halves of the molecule 82.48 (5)°. There is 1/2 molecule per asymmetric unit with a crystallographic twofold rotation axis passing through the central carbon (symmetry code: -*x*, *y*, 1/2 - *z*) of the five carbon atoms in the (—CH=N—O—(CH_2_)_5_—O—N═CH—) bridge. This structure is similar to what was observed in our previously reported salen-type bisoxime compound (Duan *et al.*, 2007). The dihedral angle formed by the two benzene rings in each molecule of the title compound is 84.18 (4)°. There are strong intramolecular O—H···N and O—H···O hydrogen bonds and weak intermolecular π–π stacking interactions between the neighbouring benzene rings, and the inter-molecular plane-to-plane distances are 3.488 (2) and 3.841 (3) Å along *b* and *c* axis, respectively. In the crystal structure, intermolecular O—H···O hydrogen bonds link each molecule to 2 others into infinite three-dimensional supramolecular structure, which is the crystal structure firstly reported of salen-type bisoxime compounds containing pentamethene bridge.

### Experimental

6,6'-Dihydroxy-2,2'-[(pentane-1,5-diyldioxy)bis(nitrilomethylidyne)]diphenol was synthesized according to an analogous method reported earlier (Dong & Feng, 2006; Dong, He *et al.*, 2008). To an ethanol solution (3 ml) of 2,3-dihydroxybenzaldehyde (138.4 mg, 1.0 mmol) was added an ethanol solution (2 ml) of 1,5-bis(aminooxy)pentane (67.4 mg, 0.5 mmol). The reaction mixture was stirred at 328 K for 8 h. After cooling to room temperature, the formed precipitate was separated by filtration, and washed successively with ethanol and ethanol–hexane (1:4), respectively. The product was dried under vacuum to yield 109.3 mg of the title compound. Yield, 58.4%. mp. 408.5–409.5 K. Anal. Calc. for C_19_H_22_N_2_O_6_: C, 60.95; H, 5.92; N, 7.48. Found: C, 60.75; H, 5.99; N, 7.42.

Pale-brown needle-like single crystals suitable for X-ray diffraction studies were obtained after two weeks by slow evaporation from a ethanol–chloroform mixed solution of the title compound.

### Refinement

Non-H atoms were refined anisotropically. H atoms were treated as riding atoms with distances C—H = 0.97 (CH_2_), or 0.93 Å (CH), O—H = 0.82 Å, and *U*_iso_(H) = 1.2*U*_eq_(C) and 1.5*U*_eq_(O).

### Crystal data

**Table:**

C_19_H_22_N_2_O_6_	*F*_000_ = 792
*M**_r_* = 374.39	*D*_x_ = 1.343 Mg m^−^^3^
Monoclinic, *C*2/*c*	Mo *K*α radiation λ = 0.71073 Å
Hall symbol: -C 2yc	Cell parameters from 875 reflections
*a* = 28.439 (3) Å	θ = 2.9–22.4º
*b* = 4.6997 (6) Å	µ = 0.10 mm^−^^1^
*c* = 14.0843 (17) Å	*T* = 298 (2) K
β = 100.354 (2)º	Needle-like, pale-brown
*V* = 1851.8 (4) Å^3^	0.46 × 0.27 × 0.25 mm
*Z* = 4	

### Data collection

**Table:**

Siemens SMART 1000 CCD area-detector diffractometer	1621 independent reflections
Radiation source: fine-focus sealed tube	837 reflections with *I* > 2σ(*I*)
Monochromator: graphite	*R*_int_ = 0.062
*T* = 298(2) K	θ_max_ = 25.0º
φ and ω scans	θ_min_ = 1.5º
Absorption correction: multi-scan(SADABS; Sheldrick, 1996)	*h* = −33→30
*T*_min_ = 0.955, *T*_max_ = 0.975	*k* = −5→5
4246 measured reflections	*l* = −16→16

### Refinement

**Table:**

Refinement on *F*^2^	Secondary atom site location: difference Fourier map
Least-squares matrix: full	Hydrogen site location: inferred from neighbouring sites
*R*[*F*^2^ > 2σ(*F*^2^)] = 0.049	H-atom parameters constrained
*wR*(*F*^2^) = 0.135	*w* = 1/[σ^2^(*F*_o_^2^) + (0.0407*P*)^2^ + 1.5785*P*] where *P* = (*F*_o_^2^ + 2*F*_c_^2^)/3
*S* = 1.00	(Δ/σ)_max_ < 0.001
1621 reflections	Δρ_max_ = 0.24 e Å^−^^3^
123 parameters	Δρ_min_ = −0.24 e Å^−^^3^
Primary atom site location: structure-invariant direct methods	Extinction correction: none

### Special details

**Table:**

Geometry. All e.s.d.'s (except the e.s.d. in the dihedral angle between two l.s. planes) are estimated using the full covariance matrix. The cell e.s.d.'s are taken into account individually in the estimation of e.s.d.'s in distances, angles and torsion angles; correlations between e.s.d.'s in cell parameters are only used when they are defined by crystal symmetry. An approximate (isotropic) treatment of cell e.s.d.'s is used for estimating e.s.d.'s involving l.s. planes.
Refinement. Refinement of *F*^2^ against ALL reflections. The weighted *R*-factor *wR* and goodness of fit *S* are based on *F*^2^, conventional *R*-factors *R* are based on *F*, with *F* set to zero for negative *F*^2^. The threshold expression of *F*^2^ > σ(*F*^2^) is used only for calculating *R*-factors(gt) *etc*. and is not relevant to the choice of reflections for refinement. *R*-factors based on *F*^2^ are statistically about twice as large as those based on *F*, and *R*- factors based on ALL data will be even larger.

### Fractional atomic coordinates and isotropic or equivalent isotropic displacement parameters (Å^2^)

**Table:**

	*x*	*y*	*z*	*U*_iso_*/*U*_eq_	Occ. (<1)
N1	0.40208 (8)	−0.0702 (5)	0.51037 (15)	0.0479 (7)	
O1	0.41975 (7)	−0.2344 (4)	0.59236 (12)	0.0542 (6)	
O2	0.39611 (7)	0.1384 (5)	0.33535 (13)	0.0637 (7)	
H2	0.4076	0.0385	0.3815	0.095*	
O3	0.34987 (8)	0.4908 (5)	0.19941 (14)	0.0782 (8)	
H3	0.3730	0.3870	0.1991	0.117*	
C1	0.46067 (10)	−0.3889 (7)	0.57460 (19)	0.0535 (9)	
H1A	0.4849	−0.2576	0.5610	0.064*	
H1B	0.4518	−0.5126	0.5192	0.064*	
C2	0.47987 (11)	−0.5624 (7)	0.66243 (18)	0.0543 (9)	
H2A	0.5048	−0.6868	0.6478	0.065*	
H2B	0.4544	−0.6814	0.6780	0.065*	
C3	0.5000	−0.3833 (9)	0.7500	0.0500 (11)	
H3A	0.4749	−0.2617	0.7657	0.060*	0.50
H3B	0.5251	−0.2617	0.7343	0.060*	0.50
C4	0.36573 (10)	0.0763 (7)	0.52087 (19)	0.0470 (8)	
H4	0.3540	0.0644	0.5783	0.056*	
C5	0.34239 (9)	0.2611 (6)	0.44479 (18)	0.0418 (7)	
C6	0.35833 (10)	0.2865 (6)	0.35701 (19)	0.0439 (7)	
C7	0.33496 (11)	0.4666 (7)	0.28589 (19)	0.0509 (8)	
C8	0.29684 (11)	0.6252 (7)	0.3023 (2)	0.0573 (9)	
H8	0.2819	0.7498	0.2550	0.069*	
C9	0.28046 (11)	0.6018 (7)	0.3883 (2)	0.0590 (9)	
H9	0.2542	0.7075	0.3985	0.071*	
C10	0.30300 (10)	0.4222 (7)	0.4586 (2)	0.0538 (9)	
H10	0.2918	0.4075	0.5165	0.065*	

### Atomic displacement parameters (Å^2^)

**Table:**

	*U*^11^	*U*^22^	*U*^33^	*U*^12^	*U*^13^	*U*^23^
N1	0.0471 (15)	0.0517 (18)	0.0431 (13)	0.0017 (13)	0.0029 (11)	0.0029 (12)
O1	0.0510 (13)	0.0670 (16)	0.0443 (11)	0.0114 (12)	0.0076 (9)	0.0072 (10)
O2	0.0557 (14)	0.0800 (17)	0.0593 (13)	0.0191 (13)	0.0211 (10)	0.0113 (11)
O3	0.0804 (16)	0.099 (2)	0.0575 (13)	0.0188 (15)	0.0195 (12)	0.0206 (13)
C1	0.0520 (19)	0.056 (2)	0.0516 (17)	0.0052 (17)	0.0074 (15)	−0.0072 (15)
C2	0.058 (2)	0.049 (2)	0.0529 (17)	0.0074 (17)	0.0018 (15)	−0.0022 (15)
C3	0.044 (2)	0.049 (3)	0.055 (2)	0.000	0.0043 (19)	0.000
C4	0.0450 (18)	0.056 (2)	0.0408 (15)	−0.0017 (16)	0.0093 (13)	−0.0038 (15)
C5	0.0342 (16)	0.045 (2)	0.0447 (15)	−0.0038 (15)	0.0033 (13)	−0.0061 (13)
C6	0.0365 (16)	0.046 (2)	0.0498 (16)	−0.0021 (15)	0.0093 (13)	−0.0063 (14)
C7	0.053 (2)	0.055 (2)	0.0440 (16)	−0.0018 (17)	0.0059 (14)	0.0002 (15)
C8	0.054 (2)	0.055 (2)	0.0579 (19)	0.0044 (18)	−0.0037 (16)	0.0007 (16)
C9	0.0465 (19)	0.063 (2)	0.066 (2)	0.0112 (17)	0.0040 (16)	−0.0084 (18)
C10	0.0471 (19)	0.062 (2)	0.0521 (17)	0.0012 (17)	0.0099 (14)	−0.0109 (16)

### Geometric parameters (Å, °)

**Table:**

N1—C4	1.273 (3)	C3—H3A	0.9700
N1—O1	1.405 (3)	C3—H3B	0.9700
O1—C1	1.431 (3)	C4—C5	1.444 (4)
O2—C6	1.360 (3)	C4—H4	0.9300
O2—H2	0.8200	C5—C10	1.394 (4)
O3—C7	1.364 (3)	C5—C6	1.396 (3)
O3—H3	0.8200	C6—C7	1.386 (4)
C1—C2	1.500 (4)	C7—C8	1.369 (4)
C1—H1A	0.9700	C8—C9	1.378 (4)
C1—H1B	0.9700	C8—H8	0.9300
C2—C3	1.518 (4)	C9—C10	1.370 (4)
C2—H2A	0.9700	C9—H9	0.9300
C2—H2B	0.9700	C10—H10	0.9300
C3—C2^i^	1.518 (4)		
			
C4—N1—O1	112.3 (2)	N1—C4—C5	120.9 (3)
N1—O1—C1	108.47 (19)	N1—C4—H4	119.5
C6—O2—H2	109.5	C5—C4—H4	119.5
C7—O3—H3	109.5	C10—C5—C6	118.3 (3)
O1—C1—C2	108.6 (2)	C10—C5—C4	119.8 (3)
O1—C1—H1A	110.0	C6—C5—C4	122.0 (3)
C2—C1—H1A	110.0	O2—C6—C7	116.5 (3)
O1—C1—H1B	110.0	O2—C6—C5	123.4 (3)
C2—C1—H1B	110.0	C7—C6—C5	120.2 (3)
H1A—C1—H1B	108.4	O3—C7—C8	119.2 (3)
C1—C2—C3	113.4 (3)	O3—C7—C6	120.7 (3)
C1—C2—H2A	108.9	C8—C7—C6	120.1 (3)
C3—C2—H2A	108.9	C7—C8—C9	120.5 (3)
C1—C2—H2B	108.9	C7—C8—H8	119.8
C3—C2—H2B	108.9	C9—C8—H8	119.8
H2A—C2—H2B	107.7	C10—C9—C8	119.8 (3)
C2—C3—C2^i^	112.6 (4)	C10—C9—H9	120.1
C2—C3—H3A	109.1	C8—C9—H9	120.1
C2^i^—C3—H3A	109.1	C9—C10—C5	121.2 (3)
C2—C3—H3B	109.1	C9—C10—H10	119.4
C2^i^—C3—H3B	109.1	C5—C10—H10	119.4
H3A—C3—H3B	107.8		
			
C4—N1—O1—C1	−179.4 (2)	O2—C6—C7—O3	−0.2 (4)
N1—O1—C1—C2	179.5 (2)	C5—C6—C7—O3	−179.2 (3)
O1—C1—C2—C3	−66.3 (3)	O2—C6—C7—C8	−179.4 (3)
C1—C2—C3—C2^i^	−178.9 (3)	C5—C6—C7—C8	1.5 (4)
O1—N1—C4—C5	179.6 (2)	O3—C7—C8—C9	178.9 (3)
N1—C4—C5—C10	−179.2 (3)	C6—C7—C8—C9	−1.9 (5)
N1—C4—C5—C6	0.2 (4)	C7—C8—C9—C10	1.2 (5)
C10—C5—C6—O2	−179.5 (2)	C8—C9—C10—C5	−0.1 (5)
C4—C5—C6—O2	1.1 (4)	C6—C5—C10—C9	−0.2 (4)
C10—C5—C6—C7	−0.5 (4)	C4—C5—C10—C9	179.2 (3)
C4—C5—C6—C7	−179.9 (3)		

Symmetry codes: (i) −*x*+1, *y*, −*z*+3/2.

### Hydrogen-bond geometry (Å, °)

**Table:**

*D*—H···*A*	*D*—H	H···*A*	*D*···*A*	*D*—H···*A*
O2—H2···N1	0.82	1.92	2.630 (3)	144
O3—H3···O2	0.82	2.24	2.689 (3)	115
O3—H3···O1^ii^	0.82	2.29	2.958 (3)	139

Symmetry codes: (ii) *x*, −*y*, *z*−1/2.
